# Retraction Note: Pets at work: integrating pet-friendly initiatives into human resources for enhanced workplace harmony

**DOI:** 10.1186/s40359-026-04466-w

**Published:** 2026-04-09

**Authors:** Ana Junça-Silva, Marisa Galrito

**Affiliations:** 1 Instituto Universitário de Lisboa (ISCTE-IUL), Lisboa, Portugal; 2 Business Research Unit – BRU (UNIDE-IUL), Lisboa, Portugal

**Retraction Note: BMC Psychology 12, 374 (2024)**

**https://doi.org/10.1186/s40359-024-01854-y**

The Editors have retracted this article after concerns were raised about its validity. A post-publication review identified issues with the measurement of basic psychological needs. The validated Work-related Basic Need Satisfaction scale requires six items per subscale, yet the authors used only two items for each construct without justification and failed to provide an explanation. This deviation does not meet accepted psychometric standards and undermines the validity of the data and the reliability of the study’s conclusions. Therefore, the Editors no longer have confidence in the results and conclusions of this article.

The authors disagree with this retraction.

